# Supplementary figures and images for: Effectiveness of a LED flashlight technique in reducing livestock depredation by lions (Panthera leo) around Nairobi National Park, Kenya
Source: PLoS One. 2018 Jan 31;13(1):e0190898. doi: 10.1371/journal.pone.0190898 (PMC5791975; doi:10.1371/journal.pone.0190898)

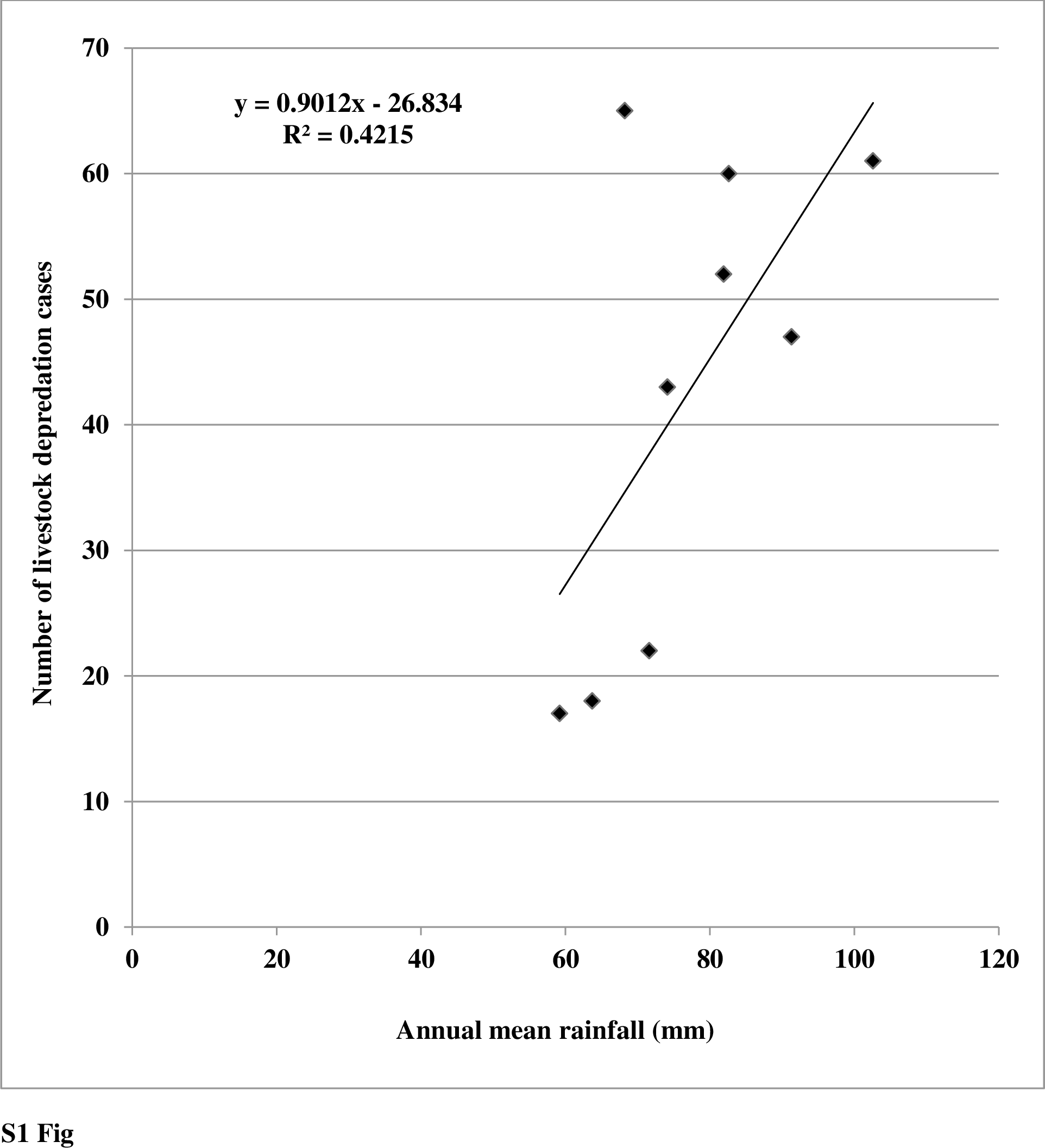

Supplement: S1 Fig — The higher the rainfall, the more predations. (TIF) [file pone.0190898.s001.tif]
