# Supplementary material for: Effectiveness of a LED flashlight technique in reducing livestock depredation by lions (Panthera leo) around Nairobi National Park, Kenya
Source: PLoS One. 2018 Jan 31;13(1):e0190898. doi: 10.1371/journal.pone.0190898 (PMC5791975; doi:10.1371/journal.pone.0190898)
Supplement: S1 Table — (DOCX) [file pone.0190898.s002.docx]

**S1 Table. Complementary predation defense deployed by the livestock owners at night based on the 2016 interviews.**

|  | Attacked | Not Attacked | X^2^ | Df | P -value |
| --- | --- | --- | --- | --- | --- |
| Radio | 2 | 5 | 0.01 | 1 | 0.920 |
| Fire | 7 | 12 | 0 | 1 | 1 |
| Prayers | 16 | 36 | 1.2 | 1 | 0.27 |
| Flashlight | 1 | 28 | 12.975 | 1 | 0.0003 |
| Scare Crow | 3 | 7 | 1.205 | 1 | 0.2723 |
| Noise | 15 | 35 | 0.499 | 1 | 0.479 |
| Spotlight | 2 | 6 | 2.26^e-31^ | 1 | 1 |
| Wood | 11 | 55 | 8.113 | 1 | 0.00439 |
| Wire | 11 | 47 | 0.5996 | 1 | 0.4406 |
| Acacia | 3 | 8 | 0.0846 | 1 | 0.7711 |
| Sheet | 3 | 5 | 0.8463 | 1 | 0.7711 |
