# Supplementary material for: Effectiveness of a LED flashlight technique in reducing livestock depredation by lions (Panthera leo) around Nairobi National Park, Kenya
Source: PLoS One. 2018 Jan 31;13(1):e0190898. doi: 10.1371/journal.pone.0190898 (PMC5791975; doi:10.1371/journal.pone.0190898)
Supplement: S2 Table — (DOCX) [file pone.0190898.s003.docx]

**S2 Table. The livestock herd size, number of attack and cases without attack.**

| Categories | Shoats | Attacked | No Attack | Total | Cattle | Attacked | NoAttack | Total |
| --- | --- | --- | --- | --- | --- | --- | --- | --- |
| Below average | >100 | 13 | 27 | **40** | >35 | 11 | 31 | **42** |
| AboveAverage | <101 | 4 | 19 | **23** | <36 | 6 | 15 | **21** |
