# Supplementary material for: Effectiveness of a LED flashlight technique in reducing livestock depredation by lions (Panthera leo) around Nairobi National Park, Kenya
Source: PLoS One. 2018 Jan 31;13(1):e0190898. doi: 10.1371/journal.pone.0190898 (PMC5791975; doi:10.1371/journal.pone.0190898)
Supplement: S3 Table — (DOCX) [file pone.0190898.s004.docx]

**S3 Table. Participants’ opinion on how to resolve human-lion Conflicts.**

| S/n | Measures to be taken to resolve human-lion conflicts in NNP | % on opinion |
| --- | --- | --- |
|  | Flashlights installation | **26.1** |
|  | Compensation | **22.8** |
|  | Keep lions in park | **12.0** |
|  | Prompt response by KWS | **7.6** |
|  | Stop construction in NNP | **6.5** |
|  | Proper fencing of bomas | **4.3** |
|  | Cooperation between community and KWS | **3.3** |
|  | Keep wild prey in park | **2.2** |
|  | Herding | **2.2** |
|  | Watch cattle at night | **2.2** |
|  | KWS to patrol at night in the community land | **2.2** |
|  | Translocation of problem animals | **2.2** |
|  | KWS to monitor lions | **2.2** |
|  | Reduce lion numbers in NNP | **1.1** |
|  | Monitor collared lions and bring them back to NNP | **1.1** |
|  | Train people from the community and let them monitor lions | **1.1** |
|  | Feed lions if hungry | **1.1** |
