# Supplementary material for: Effectiveness of a LED flashlight technique in reducing livestock depredation by lions (Panthera leo) around Nairobi National Park, Kenya
Source: PLoS One. 2018 Jan 31;13(1):e0190898. doi: 10.1371/journal.pone.0190898 (PMC5791975; doi:10.1371/journal.pone.0190898)
Supplement: S1 File — (DOCX) [file pone.0190898.s005.docx]

**S1 File. Final Questionnaire**

(*Additional questions of 2014 in italic)*

Date:

GPS Coordinates:

1. *Do you keep all your livestock in bomas at night, or only part of it?*

*All livestock/ part of it (____ %) / none*

1. *If none at night, where do you keep your livestock at night?*
2. *Do you keep all livestock in one boma or in several bomas?*
3. Could you describe the boma(s) construction materials and properties?

| 3 a. Can livestock be perceived through the boma structure? | Yes visible |  |
| --- | --- | --- |
|  | Partially visible |  |
|  | Not visible |  |
| 3 b. How high is the boma structure? | 0 – 1 meters |  |
|  | 1 – 2 meters |  |
|  | more than 2 meters |  |
| 3 c. What is the thickness of the boma? | 0 – 0.25 meters |  |
|  | 0.25 – 0.5 meters |  |
|  | More than 0.5 meters |  |
| 3 d. From which material is the boma constructed? | Bush (acacia) |  |
|  | Fence (chain-link/barbed) |  |
|  | Stone (stones/bricks/cement) |  |
|  | Sheets (metal/wood) |  |
|  | Wood (offcuts/posts/poles) |  |
|  | House (inside house) |  |
|  | Mixture of the above *(specify which)* |  |

1. Has your boma(s) suffered any attack over the last 2 years?

(to be filled in Appendix II)

- When (dates and time of the day)?
- Which predator was responsible for the attack?
- Who witnessed the predator?
- Which animals and how many did the predator kill?
- Did you report the attack? To whom (KWS, Area Chief, FONNAP, Game Scout)?

1. Do you think the attacks could have been prevented? How?
2. Do you have flashlights installed in your boma(s)? y/n
3. If yes, when was the flashlight installed?
4. Has there been any predation since the flashlight was installed? y/n
5. If yes to Q7, are you happy with the functioning of the flashlights? y/n
6. *Could you name what other preventive methods against livestock predation do you use at day/night?*

|  | Preventive method | Day | Night |
| --- | --- | --- | --- |
|  | Radio |  |  |
|  | Dogs |  |  |
|  | Fire |  |  |
|  | Human guards |  |  |
|  | Scare crows |  |  |
|  | Herding |  |  |
|  | Noise |  |  |
|  | Prayers |  |  |
|  | Flashlights |  |  |
|  | Others |  |  |

1. Do you think fencing the southern border of the park could help prevent lion attacks? y/n
2. In your opinion, what do you think can be done to resolve human-lion conflict in this area?
3. *Are you aware of the satellite collaring of lions by the Nairobi lion project ? y/n*
4. *What is your opinion about the satellite collaring of lions*

very good/ good/ no opinion/ bad/very bad

1. What is your main source of income?

(livestock/ farmer/ employed/ business (e.g. grocery)/ other:____________)

1. *How many livestock do you intend to keep?*
2. How many livestock do you currently have?

Name:

Age:

Sex: m/f

Education level: none/ school/ high-school/ college/ other:

Ethnicity:
